# Susceptibility of *Bemisia tabaci* Gennadius (Hemiptera: Aleyrodidae) Mediterranean Populations Found in São Paulo, Brazil to 11 Insecticides and Characterization of Their Endosymbionts

**DOI:** 10.3390/insects15090670

**Published:** 2024-09-04

**Authors:** Daniel de Lima Alvarez, Rafael Hayashida, Michael C. Cavallaro, Daniel Mariano Santos, Lucas Moraes Santos, Cristiane Müller, Luís Fernando Maranho Watanabe, Vinicius Henrique Bello, Renate Krause-Sakate, William Wyatt Hoback, Regiane Cristina de Oliveira

**Affiliations:** 1 Crop Protection Department, School of Agronomic Sciences, São Paulo State University “Júlio de Mesquita Filho” (FCA/UNESP), Botucatu 18610-034, SP, Brazil; daniel-alvarez92@hotmail.com (D.d.L.A.); d_m.s@hotmail.com (D.M.S.); renate.krause@unesp.br (R.K.-S.); regiane.cristina-oliveira@unesp.br (R.C.d.O.); 2 Department of Entomology and Plant Pathology, Oklahoma State University, Stillwater, OK 74078, USA; michael.cavallaro@okstate.edu; 3 Biotrop, Solutions in Biological Technologies, Curitiba 81460-020, PR, Brazil; santosmlucas1@gmail.com (L.M.S.); lfmwatanabe@gmail.com (L.F.M.W.); 4 Corteva Agriscience, Mogi Mirim 13814-000, SP, Brazil; cristiane.muller@corteva.com; 5 Department of Plant Pathology and Nematology, Escola Superior de Agricultura Luiz de Queiroz, University of São Paulo, Piracicaba 13418-900, SP, Brazil; vhbello@hotmail.com

**Keywords:** silverleaf whitefly, neonicotinoid, toxicity, microbiome, integrated pest management

## Abstract

**Simple Summary:**

Previous studies have documented regional susceptibility of the silverleaf whitefly (*Bemisia tabaci*) to common insecticide active ingredients among populations with different bacterial endosymbionts. Here, we tested the efficacy of 11 different insecticides on three populations of silverleaf whitefly found in Brazil with varying bacterial communities. Significant differences among population responses were measured for four of the tested active ingredients, two of which were neonicotinoids. DNA sequencing detected two types of bacteria in the population more susceptible to insecticides, showing a linkage of host, symbiote, and insecticide resistance.

**Abstract:**

The silverleaf whitefly, *Bemisia tabaci* Gennadius (Hemiptera: Aleyrodidae), is a significant agricultural pest worldwide, impacting a variety of crop yields. Since the introduction of *B. tabaci* Mediterranean (MED) species in Brazil, limited research has measured the relative efficacy of the primary insecticides used in whitefly management. This study evaluated the susceptibility of three distinct *B. tabaci* MED populations to 11 insecticide active ingredients and characterized the bacterial endosymbionts within each population. The insecticides tested were acetamiprid, bifenthrin, cyantraniliprole, diafenthiuron, spiromesifen, imidacloprid, pymetrozine, pyriproxyfen, sulfoxaflor, and thiamethoxam. Results showed varying LC_50_ and LC_90_ values among tested insecticides and populations. Notably, populations varied in response to imidacloprid and thiamethoxam with some populations having a 6× higher tolerance. Sequencing data of endosymbionts revealed that individuals from the most susceptible *B. tabaci* population harbored *Rickettsia* and *Arsenophonus*, whereas these bacteria were not detected in the resistant populations. These findings highlight the need for frequent insecticide toxicity bioassays of distinct *B. tabaci* populations and the adoption of integrated pest management strategies to preserve the efficacy of insecticides for *B. tabaci* control. Additionally, the role of infection by endosymbionts to alter susceptibility should be further explored.

## 1. Introduction

The silverleaf whitefly, *Bemisia tabaci* Gennadius (Hemiptera: Aleyrodidae), is a widespread cryptic species complex that impacts numerous agricultural and horticultural crops globally [1,2,3]. Phloem-feeding nymphs and adults damage ornamental and crop plants and cause yield loss directly by consuming sap from the plant and indirectly by generating honeydew that allows fungal growth as well as by transmitting more than 400 different plant viruses [4,5,6].

The *B. tabaci* species complex comprises as many as 48 species that cannot be distinguished morphologically [2,7,8]. Distinct cryptic species are primarily differentiated based on biochemical or molecular polymorphism markers. Additionally, *B. tabaci* exhibit varying biological characteristics, including differences in host plant preference, capacity for differential plant injury, expression of insecticide resistance, and the ability to transmit plant viruses [9,10,11]. Among the cryptic species are two biotypes, Middle East-Asia Minor 1 (MEAM1, formerly biotypes B and B2) and Mediterranean (MED, formerly biotypes Q, J, and L) species, which are considered some of the most destructive horticultural pests and are distributed worldwide [12,13].

The dynamics of *B. tabaci* species presence, abundance, and damage have likely changed as a result of frequent insecticide use and the development of insecticide resistance [14]. Currently in most regions, *B. tabaci* MED has replaced *B. tabaci* MEAM1, especially in areas exposed to high insecticide pressure—typically neonicotinoids and insect growth regulators [9,15,16,17]. The differential survivorship of *B. tabaci* MED is linked to its reduced susceptibility to insecticide molecules. The factors influencing survival include genetic characteristics [18,19], the specific mode of action and frequency of insecticides used in pest management within the area [16], as well as the composition of endosymbionts and enzymes within whitefly population [10,20,21].

Recently, the characterization of bacterial endosymbionts found in *B. tabaci* populations has increased in importance, with several studies highlighting their role in insecticide resistance [20,22,23,24]. Among the detected microbe taxa, the obligatory bacterium *Portiera aleyrodidarum* Thao & Baumann occurs in all whitefly species with secondary endosymbionts *Arsenophonus*, *Cardinium*, *Fritschea*, *Hamiltonella*, *Hemipteriphilus*, *Rickettsia* and/or *Wolbachia*, occurring in variable combinations [10,20,21]. Microbiomes from wild-type populations may harbor over 60 genera of bacteria, with some yet to be described [25,26]. Regional susceptibility to insecticide exposure, accounting for previous exposure and convenience of insecticide product application, will vary based on bacterial endosymbionts presence and absence, requiring local evaluations to best prepare farmers, agricultural extension, and IPM practitioners.

In Brazil, *B. tabaci* MEAM1 was introduced into Sao Paulo State in the 1990s on ornamental plants [27]. In 2015, *B. tabaci* MED was first detected in Rio Grande do Sul and has spread to São Paulo and other parts of Brazil [28]. Given the increasing importance of *B. tabaci* MED in Brazil following its introduction [24,28] and the limited knowledge regarding this species’ susceptibility to the primary insecticides used in whitefly management, it becomes crucial to measure the efficacy of commonly used insecticides in Brazil and compare among populations. Additionally, the quantification and identification of bacterial endosymbionts within each population would provide insight to the potential variability among distinct MED populations. Therefore, the objective of our current study was specific to whitefly management in Brazil and two-fold: first, to determine the LC_50_ and LC_90_ values (lethal concentration causing 50 and 90% mortality) for the main insecticides used in *B. tabaci* management among three distinct MED populations; and second, to identify the frequency of endosymbionts within each of these populations.

## 2. Materials and Methods

### 2.1. Whitefly Laboratory Rearing

Three *B. tabaci* MED populations were originally collected from different crop fields across three counties in São Paulo state. In August 2018, the population from São Pedro do Turvo (SPT) municipality was collected in a commercial bell pepper (*Capsicum annuum*) field (22°47′10″ S 49°51′17″ W). Meanwhile, the population from Holambra (HL; 22°37′59″ S 47°03′20″ W) was collected from a Hibiscus tree (*Hibiscus* spp.) in July 2017, and the Santa Isabel (SI; 23°22′20″ S 46°10′35″ W) population was obtained from *Begonia* spp. in August 2018. These insects were housed in cages (45 × 45 × 55 cm) and reared on bell pepper plants in a climate-controlled environment at 26 ± 2 °C, with a relative humidity of 80 ± 10%, and a photoperiod of 14:10 h (L:D). Adult whiteflies from each population were randomly collected and placed in Eppendorf^®^ (Eppendorf, Enfield, CT, USA) tubes containing 95% ethanol and then stored at −20 °C for subsequent analysis.

### 2.2. B. tabaci MED Identification

To genetically identify each population, we performed an analysis of mitochondrial cytochrome oxidase subunit I (mtCOI) gene sequences. Initially, DNA extraction was carried out on ten individuals from each population using the Chelex protocol [29], and the samples were PCR amplified using the primers C1-J-2195 and TL2-N-3014 [30].

The PCR reaction were carried out in a final volume of 50 µL (with a final concentration of 50 mM MgCl_2_, 2.5 mM dNTPs, and 1 µM oligonucleotides) using 0.5 units of Taq polymerase. The reaction followed a cycle of 5 min at 94 °C, 30 s at 94 °C, 45 s at 45 °C, and 1 min at 72 °C (for 35 cycles) with a final extension of 10 min at 72 °C. Subsequently, polymorphism analysis by RFLP (Restriction Fragment Length Polymorphism) [31] allowed differentiation of the MEAM1, MED, NW1, and NW2 species. The sequenced nucleotides were analyzed and compared with other deposited whitefly sequences in GenBank (GenBank access: KX673609).

### 2.3. Endosymbionts Identification

The DNA samples extracted from 100 individuals per population were screened for *Portiera aleyrodidarum*, the known primary endosymbiont of *B. tabaci*, as well as six secondary endosymbionts: *Hamiltonella*, *Rickettsia*, *Wolbachia*, *Arsenophonus*, *Cardinium*, and *Fritschea*, which have been reported in whiteflies. We used genus-specific primers targeting the 16S or 23S rDNA genes. PCR cycling followed the protocol described by Marubayashi et al. [32] and Moraes et al. [24]. To confirm the presence of endosymbionts, we sequenced the amplified sequences from representative individuals.

### 2.4. Insecticides

The commercially formulated insecticide products used for the toxicity bioassays, each with the content of the corresponding active ingredient (a.i.) were Closer 240 SC (a.i.: sulfoxaflor 240 g ai L^−1^) Dow AgroSciences Industrial Ltd.a., Brazil; Provado 200 SC (a.i.: imidacloprid 200 g ai L^−1^) Bayer AG, Germany; Mospilan 200 SP (a.i.: acetamiprid 200 g ai L^−1^) Iharabras SA, Brazil; Actara 250 WG (a.i.: thiamethoxam 250 g ai L^−1^), Syngenta Ltd.a., Brazil; Benevia 100 OD (a.i.: cyantraniliprole 100 g ai L^−1^) FMC Chemicals, South Africa; Chess 500 WG (a.i.: pymetrozine 500 g ai L^−1^) Syngenta, Brazil; Polo 500 SC (a.i.: diafenthiuron 500 g ai L^−1^) Syngenta, Brazil; Talstar 100 EC (a.i.: bifenthrin 100 g ai L^−1^) FMC Química, Brazil; Orthene 750 BR (a.i.: acephate 750 g ai L^−1^) UPL, Brazil; Tiger 100 EC (a.i.: pyriproxyfen 100 g ai L^−1^) Sumitomo Chemical; Brazil and Oberon (a.i.: spiromesifen 240 g ai L^−1^) Bayer AG, Germany.

### 2.5. Bioassays

Bioassays were conducted using second instar nymphs of MED whiteflies. Initially, 10 adults were placed in mini cages made of metal clips and plastic tubes (clip-cages) on tomato (*Solanum lycopersicum* L.) plants approximately 30 days after transplantation. Adults remained in cages for 48 h for oviposition. Plants were kept under controlled conditions at 26 ± 2 °C, with a relative humidity of 80 ± 10% and a 14 h photoperiod, until the eggs hatched and the nymphs reached the second instar. Using non-toxic acrylic glue and a magnifying glass, 20 nymphs per leaflet were glued [33].

Initially, 10 different concentrations were tested for each insecticide. Of these, 6 to 8 suitable concentrations were adopted for the LC_50_ and LC_90_ curves. Dilutions for each concentration were made from a stock solution with a concentration of 1% active ingredient of the commercial product following the IRAC protocol [34]. A total of 120 nymphs per plant were tested, with 6 replicates, and each tested plant represented a concentration series. To perform the test, the leaflets carrying 20 nymphs were immersed in their respective concentrations for 5 s, including a control group immersed in water. Plants containing the tested nymphs remained in a controlled environment for approximately 9 days.

### 2.6. Statistical Analysis

Bioassay data were analyzed by probit using POLO PLUS software 1.0 [35], and the figures were designed using Prism software 10.0 [36]. The software tests the linearity of dose–mortality response and provides the slope, the 50 and 90% lethal concentrations (LC_50_ and LC_90_) and the 95% confidence limits of the LC for each mortality line. Mortality was determined by the difference between the total number of nymphs per replicate and the total number of nymphs that reached the 4th instar. The LC_50_ and LC_90_ values of insecticides were considered significantly different when their 95% confidence limits did not overlap.

## 3. Results

The genetic identity of each population collected in the field was confirmed through PCR analysis, revealing that all three populations were MED populations.

In our current study, the LC_50_ and LC_90_ values varied among the tested insecticides and among MED populations (Table 1). The lowest LC_50_ was observed for imidacloprid in the HL population, with a concentration of 4.52 mg/L, while the highest LC_50_ was associated with thiamethoxam in the SPT population, reaching 142.84 mg/L. As for the LC_90_, cyantraniliprole exhibited the lowest value in the SI population (57.34 mg/L), whereas thiamethoxam had the highest LC_90_ in the SPT population, at 1362.0 mg/L (Table 1).

Significant differences in LC_50_ among populations were found for imidacloprid, thiamethoxam, pyriproxyfen, and diafenthiuron; while for LC_90_, the only difference among populations was found for thiamethoxam (Table 1). Interestingly, in all these cases, the HL population consistently exhibited the lowest LC_50_ and LC_90_ values.

**Figure 1:**
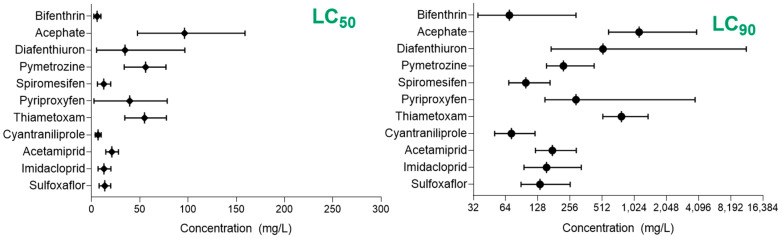
Combined analysis of lethal concentrations for three different *Bemisia tabaci* MED populations and its 95% confident limits. LC_50_ = lethal concentrations of insecticide killing 50% of the population; and LC_90 =_ lethal concentrations of insecticide killing 90% of the population.

The combined analysis indicated that despite differences among MED populations tested, bifenthrin exhibited the lowest LC_50_ (5.73 mg/L), while acephate had the highest (96.37 mg/L). The lowest LC_90_ was observed for bifenthrin (69.41 mg/L), whereas acephate had the highest (1137.3 mg/L; Figure 1).

The PCR analysis revealed that the frequency of endosymbionts in the SPT population was 80% for individuals infected with *Hamiltonella* and 100% for those infected with *Wolbachia* (Table 2). In the HL population, all individuals harbored *Hamiltonella*, while 10% harbored *Rickettsia*, 10% harbored *Wolbachia* and 10% harbored *Arsenophonus*. Lastly, the SL population had 97% of its individuals infected with *Hamiltonella*. The endosymbionts *Cardinium* and *Fritschea* were not detected in any of the populations tested.

## 4. Discussion

Worldwide, varying levels of insecticide susceptibility are reported in regional *B. tabaci* populations, depending on the *B. tabaci* cryptic species, strain, life stage, and endosymbionts present [20,22,37,38,39,40]. Within the last decade, *B. tabaci* MED has developed resistance to various insecticides classes, including the novel ones [14]. Thus, the frequent and recurrent evaluation of the susceptibility of different populations is necessary to potentially delay or even prevent the emergence of resistant populations of *B. tabaci* MED to insecticides.

Here, in the HL population, the LC_50_ data for imidacloprid, thiamethoxam, pyriproxyfen, and diafenthiuron were significantly different than the least susceptible tested population (SPT and SI). Overall, the HL population was the most sensitive group in 7 of the 11 insecticides tested. Among the insecticides that displayed higher toxicity to the HL population, imidacloprid and thiamethoxam measured the greatest difference from the least susceptible population with a 6-fold increase in sensitivity (LC_50_ data). The average difference in sensitivity between the HL population and least susceptible population for the seven insecticides was 3.7-fold (LC_50_ data). Previous research also reported different LC_50_ values for neonicotinoids among localities [41]. Frequent assessments of *B. tabaci* population susceptibility to neonicotinoids, along with historical records of insecticide use, are valuable and can better inform future management decisions. Neonicotinoids are widely adopted in whitefly management due to their exceptional effectiveness [42]. However, there is a strong correlation between the frequency of neonicotinoid applications in the field and reduced efficacy [41], and MED is frequently related to high resistance and cross-resistance to neonicotinoids [9,43]. Moreover, Barman et al. [44] identified upregulated P450 genes in *B. tabaci* populations associated with resistance to imidacloprid and thiamethoxam, suggesting a more complex relationship between distinct *B. tabaci* populations and neonicotinoid resistance. Future work should examine the gene upregulation of known neonicotinoid detoxification enzymes in concert with other factors that influence resistance (i.e., bacterial endosymbionts).

Similar to previous documented cases of differential susceptibility, the observed response among MED populations might be explained by the endosymbiont composition of each population. PCR identified the genera *Rickettsia* and *Arsenophonus* only from HL populations. Previous work with MED (Q biotype) reported a higher sensitivity to insecticide exposure doubly infected with *Rickettsia–Arsenophonus* [20]. Of further interest, *Rickettsia* is not only transmitted vertically between generations but also can be horizontally transferred between males and females during mating, despite the potential deleterious fitness cost [8,45,46], which can potentially be useful for future *B. tabaci* management strategies. *Rickettsia* will colonize most tissues and organs in whiteflies, unlike some other bacterial endosymbionts which are restricted to the bacteriosome [20]. Kontsealov et al. [10] found a significant increase in susceptibility to acetamiprid, thiamethoxam, spiromesifen, and pyriproxyfen exposure in *Rickettsia*-inoculated lines of *B. tabaci* from a laboratory culture with known bacterial endosymbionts. Due to the unique role of *Rickettsia* in whiteflies, increased susceptibility to insecticide exposure has also been documented for different life stages. For example, populations infected with *Rickettsia* exhibited a significant increase in egg mortality when exposed to pyriproxyfen [8]. Documented susceptibility among tested insecticides increases regardless of molecular target sites and mode of action. Although the mechanisms underlying the interactions between *B. tabaci* and endosymbionts for insecticide resistance are not completely clear [22], more studies are required to explore the endosymbiont-based approach for *B. tabaci* using *Rickettsia* associated with chemical insecticides. Observed congruences among our data and previous work indicates that the relationship between Rickettsia and insecticide susceptibility is possibly widespread among different populations of *B. tabaci.*

The relationship between insecticide resistance and the presence of *Wolbachia* bacteria in insects is well established [22,47,48], and *Wolbachia* infections in *B. tabaci* populations have been measured worldwide [49]. Here, PCR detected *Wolbachia* in 10% of the individuals from the HL population and 100% of the individuals from the SPT population (Table 2). Corresponding with this difference in endosymbionts, the SPT population was the least sensitive group in 5 of 11 insecticides tested. As mentioned, the greatest difference in LC_50_ values among the tested insecticides was measured in imidacloprid and thiamethoxam. Barman et al. [44] found a significant positive correlation between an increased presence of *Wolbachia* and resistance to imidacloprid, thiamethoxam, and acetamiprid. This is consistent with Ghanim and Kontsedalov [20], which determined that the presence of *Wolbachia* increased the susceptibility of *B. tabaci* to imidacloprid, thiamethoxam, and pyriproxyfen. Moreover, the increased density of bacterial endosymbionts likely provides the host insect with a more diverse array of detoxification mechanisms, i.e., the expression of P450 monooxygenases [20,44]. Interestingly, a recent study found that a minimal divergence in genetic variation is observed among *B. tabaci* populations found in Asia, suggesting that the acquisition of secondary endosymbionts (i.e., *Rickettsia* and *Wolbachia*) is regional [44]. This adds to the importance of characterizing the local endosymbiont dynamics among *B. tabaci* populations. 

In insects, bacterial endosymbionts play a variable but important role in protecting hosts from environmental stress, including insecticide exposure [22]. Here, the presence of the nonessential, facultative bacterial endosymbionts *Wolbachia* and *Rickettsia* influenced insecticide susceptibility among distinct MED populations. Both *Rickettsia* and *Wolbachia* are widely documented among different populations and species of whitefly species found in Brazil [32]. Molecular tools paired with toxicity assessments can continue to optimize regional susceptibility.

Commercially formulated insecticide products are currently the main approach adopted for *B. tabaci* control, and due to known nontarget environmental concerns and the development of resistance, the reliance on convenient insecticide application can be considered troublesome [14]. Future whitefly management tactics should adopt all available IPM methods harmoniously to preserve the efficacy of the available insecticide products for *B. tabaci* control. Regional insecticide susceptibility data of three different MED populations from Brazil paired with endosymbionts composition will be valuable information for local farmers, agricultural extension, and IPM practitioners.

## Figures and Tables

**Table 1:** Sensitivity of second instar nymphs of three *B. tabaci* MED populations to different insecticides (a.i.).

Insecticide	Population	*n*	Slope (±SE ^1^)	LC_50_ (mg/L) (95% CL) ^2^	LC_90_ (mg/L) (95% CL) ^3^	X^2^ (DF) ^4^
Acephate	SPT	840	1.38 (0.11)	120.12 (68.50–186.29) ^ns^	1005.32 (573.12–2628.65) ^ns^	8.57 (4)
	HL	840	1.10 (0.10)	53.34 (14.33–103.60)	764.98 (353.70–4727.30)	13.17 (4)
	SI	840	1.14 (0.11)	132.83 (38.42–286.19)	1739.7 (665.07–27219.0)	17.17 (4)
Acetamiprid	SPT	720	1.79 (0.19)	18.88 (1.71–35.16) ^ns^	97.64 (53.60–778.81) ^ns^	13.11 (3)
	HL	960	1.10 (0.08)	25.73 (8.30–64.21)	374.84 (123.81–9150.2)	34.77 (5)
	SI	840	1.35 (0.12)	20.19 (11.41–29.67)	179.31 (110.55–415.44)	6.43 (4)
Bifenthrin	SPT	840	1.24 (0.11)	5.70 (1.98–10.29) ^ns^	61.10 (30.32–290.95) ^ns^	12.70 (4)
	HL	960	1.19 (0.09)	24.26 (7.91–58.36)	285.07 (101.91–5012.8)	37.79 (5)
	SI	840	1.21 (0.11)	5.41 (1.65–10.16)	61.38 (29.27–356.95)	13.97 (4)
Cyantraniliprole	SPT	960	1.23 (0.13)	5.30 (3.28–7.50) ^ns^	57.67 (43.33–83.10) ^ns^	2.98 (5)
	HL	840	1.21 (0.14)	8.68 (1.83–17.39)	99.59 (49.59–479.12)	9.34 (4)
	SI	840	1.33 (0.14)	6.25 (1.43–12.29)	57.34 (30.38–201.56)	10.88 (4)
Diafenthiuron	SPT	840	1.82 (0.13)	54.91 (31.16–85.71) b	275.77 (164.82–673.62) ^ns^	13.71 (4)
	HL	840	1.84 (0.15)	14.35 (3.81–27.41) a	71.01 (36.33–390.42)	25.22 (4)
	SI	960	1.68 (0.12)	29.55 (19.26–41.57) ab	169.54 (114.13–301.76)	9.53 (5)
Imidacloprid	SPT	840	1.67 (0.14)	27.21 (6.82–53.30) ab	158.67 (78.89–877.39) ^ns^	23.52 (4)
	HL	840	0.86 (0.10)	4.52 (1.42–8.50) a	137.13 (59.47–852.19)	6.97 (4)
	SI	840	1.62 (0.14)	16.79 (12.82–21.10) b	103.53 (79.91–143.47)	3.34 (4)
Pymetrozine	SPT	840	3.34 (0.34)	67.85 (38.01–91.06) ^ns^	163.93 (120.30–329.46) ^ns^	13.50 (4)
	HL	720	1.49 (0.17)	45.68 (14.98–75.99)	329.51 (173.34–2263.74)	7.35 (3)
	SI	840	2.16 (0.23)	55.13 (30.50–76.69)	215.92 (152.92–412.86)	7.49 (4)
Pyriproxyfen	SPT	1080	1.85 (0.14)	57.65 (42.35–73.46) b	282.55 (213.27–418.11) ^ns^	8.50 (6)
	HL	840	1.06 (0.11)	12.20 (2.43–26.13) a	195.17 (83.92–1546.45)	12.82 (4)
	SI	840	1.95 (0.15)	45.00 (26.11–66.22) ab	204.01 (132.38–423.39)	10.87 (4)
Spiromesifen	SPT	840	1.48 (0.17)	14.17 (2.47–27.18) ^ns^	103.95 (66.35–216.14) ^ns^	9.50 (4)
	HL	840	1.51 (0.14)	12.09 (1.86–25.33)	85.10 (39.57–789.17)	23.52 (4)
	SI	840	1.73 (0.18)	18.19 (3.36–33.44)	99.35 (61.53–220.69)	13.24 (4)
Sulfoxaflor	SPT	840	1.13 (0.11)	13.44 (5.32–22.44) ^ns^	182.06 (100.40–597.69) ^ns^	7.87 (4)
	HL	840	1.54 (0.15)	14.59 (6.20–23.082)	99.10 (61.72–246.36)	9.11 (4)
	SI	840	1.30 (0.12)	13.56 (6.84–20.78)	130.82 (82.35–285.68)	5.98 (4)
Thiamethoxam	SPT	840	1.30 (0.14)	142.84 (70.85–230.92) b	1362.00 (732.33–4552.6) b	6.64 (4)
	HL	960	1.09 (0.09)	24.33 (13.02–37.69) a	358.22 (228.14–669.58) a	6.97 (5)
	SI	840	1.34 (0.13)	57.33 (17.58–103.20) b	512.87 (275.62–1961.23) ab	11.84 (4)

^1^ ±1 Standard error. ^2^ Lethal concentrations of insecticide killing 50% of population and its 95% confidence limits. ^3^ Lethal concentrations of insecticide killing 95% of population and its 95% confidence limits. ^4^ Chi-square testing linearity of dose-mortality responses; DF = degrees of freedom. Means followed by different letters (±SE) indicate statistical differences. ^ns^ = non-significant differences.

**Table 2:** Infection frequencies (%) of secondary endosymbionts in the *Bemisia tabaci* MED population.

Population	Frequency of Individuals with Endosymbionts (%)					
	*Hamiltonella*	*Rickettsia*	*Wolbachia*	*Cardinium*	*Arsenophonus*	*Fritschea*
SPT	80	0	100	0	0	0
HL	100	10	10	0	10	0
SI	97	0	0	0	0	0

## Data Availability

The original contributions presented in the study are included in the article, further inquiries can be directed to the corresponding authors.
